# Masticadienonic and 3α-OH Masticadienoic Acids Induce Apoptosis and Inhibit Cell Proliferation and Tumor Growth in Prostate Cancer Xenografts In Vivo

**DOI:** 10.3390/molecules22091479

**Published:** 2017-09-06

**Authors:** Ma. Beatriz Sánchez-Monroy, Nadia J. Jacobo-Herrera, Alejandro Zentella-Dehesa, Beatriz Hernández-Téllez, Mariano Martínez-Vázquez

**Affiliations:** 1 Departamento de Productos Naturales, Instituto de Química, Universidad Nacional Autónoma de México, Circuito Exterior, Ciudad Universitaria, Delegación Coyoacán C.P., CDMX 04510, Mexico; beatriz.sanchez@ciencias.unam.mx; 2 Departamento de Bioquímica, Instituto Nacional de Ciencias Médicas y Nutrición Salvador Zubirán, Vasco de Quiroga 15, Sección XVI, Delegación Tlalpan C.P., CDMX 14000, Mexico; nadia.jacobo@gmail.com (N.J.J.-H.); azentell@iibiomedicas.unam.mx (A.Z.-D.); 3 Departamento de Biología Celular y Tisular, Facultad de Medicina, Universidad Nacional Autónoma de México, Circuito Exterior, Ciudad Universitaria, Delegación Coyoacán C.P., CDMX 04510, Mexico; bhernandezt@hotmail.com; 4 Departamento de Medicina Genómica y Toxicología Ambiental & Programa Institucional de Cáncer de Mama, Instituto de Investigaciones Biomédicas, Universidad Nacional Autónoma de México, Circuito Deportivo, Ciudad Universitaria, Delegación Coyoacán C.P., CDMX 04510, Mexico

**Keywords:** masticadienonic acid, 3α-OH masticadienonic acid, prostate cancer, xenografts, PCNA, Ki-67, apoptosis

## Abstract

The triterpenes have been constituted as a group of interesting molecules as possible antitumor agents. Despite several of them not presenting a potent cytotoxic activity in vitro against cancer cells, in vivo in xenotransplant tumors studies, they show promising results. Based on the above considerations, we investigated the antitumor activity of both masticadienonic (MDA) and 3α-OH masticadienoic (3α-OH MDA) acids in a mouse prostate cancer xenograft model. Immunohistochemical assays were used to evaluate the decrease in the expression of the Proliferating Cell Nuclear Antigen (PCNA) and the Ki-67 induced by MDA and 3α-OH MDA. Terminal deoxynucleotidyl transferase dUTP nick end labeling (TUNEL) assay was performed to demonstrate the fragmentation of DNA. Our results showed that the two triterpenes inhibited tumor growth, had anti-proliferative effect in vivo and induced cell death by apoptosis. Collectively, our data suggested that the antitumor mechanism of MDA and 3α-OH MDA involves several molecular targets related to cell proliferation and apoptosis.

## 1. Introduction

Prostate cancer (PC) is the second leading cause of cancer death in men, accounting for approximately 27,540 deaths worldwide in 2015 [1]. PC begins as an androgen-dependent disease; the standard treatment options usually include surgery, radiation, active surveillance and androgen-deprivation therapy. Although these approaches are initially positive, 25 to 40 percent of the patients progress to an advanced stage [2,3]. In this stage, 50 to 70% of the patients receive chemotherapy resulting in progression-free survival but without curative results [4,5]. Further efforts are needed to investigate novel treatments for incurable PC.

Plants are a rich source of antitumor compounds; from the 1940s until now, 48.6% of the drugs approved by the Food and Drug Administration(FDA)are natural or naturally derived products [6]. In recent studies, the antitumor activities of some triterpenes, such as ursolic acid, acetyl-11-keto-beta-boswellic acid (AKBA) and 3-β-acetoxy-tirucallic acid (βATA) [7,8,9] have been reported.

**Figure 1:**
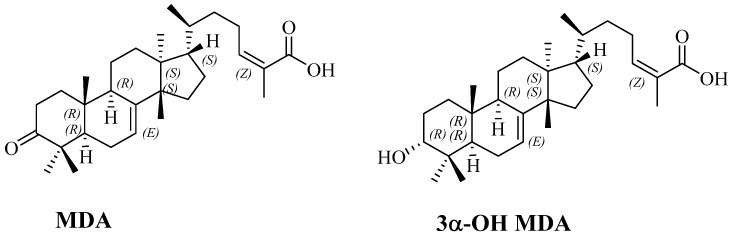
Chemical structures of masticadienonic (MDA) and 3α-hydroxymasticadienoic (3α-OH MDA) acids.

*Amphipterygium adstringens* Schiede Schlecht (common name: Cuachalalate) is a medicinal plant with a long tradition of ethnobotanical use in treating several ailments, including gastric cancer and anti-inflammatory conditions [10,11]. Previous work has shown that the major components of the bark of *A. adstringens* comprise some tirucallane-type triterpenes, such as masticadienonic acid (MDA) and 3-α-hydroxy masticadienoic acid (3α-OH MDA) (Figure 1) [12]. Previously, we demonstrated anti-proliferative and anti-inflammatory activities of MDA and 3α-OH MDA [13], and also that 3α-OH MDA impairs mitochondrial functions [14].

Based on the above findings, we decided to evaluate the antitumor activities of MDA and 3α-OH MDA acids in a mouse xenograft model.

## 2. Results

### 2.1. Acute Toxicity Test

The results indicated that only the 1000 mg/kg dose of MDA or 3α-OH MDA induced death in the animals. To determine the median lethal dose (LD_50_) of each triterpene, 250, 500 and 750 mg/kg of MDA and 170, 210, 250 and 500 mg/kg of 3α-OH MDA were used. The lethal dose 50 (LD_50_) was calculated from the square root of the product of the lowest lethal dose and the highest non-lethal dose. The LD_50_ of MDA and 3α-OH MDA were calculated from the equations $\sqrt{\left(500\times 250\right)}$ and $\sqrt{\left(170\times 210\right)}$, respectively [15]. The LD_50_ values were determined as 353.55 mg/kg and 188.94 mg/kg for MDA and 3α-OH MDA, respectively.

### 2.2. Cytotoxicity In Vitro of MDA and 3α-OH MDA on PC-3 Cells

The cytotoxic effect of masticadienonic and 3α-OH masticadienoic acids in the PC-3 cells was tested by the crystal violet colorimetric method. Cisplatin was used as the positive control. The PC-3 cells were exposed to concentration ranges of 12.5 to 100 µM of the triterpenes for 48 h. Bioactivities of MDA and 3α-OH MDA were determined from the concentration that induced 50% growth inhibition in the treated cells compared to the controls. The results are shown in Table 1.

### 2.3. Masticadienonic and 3α-Hydroxy Masticadienoic Acids Inhibited the Growth of Prostate Cancer Xenografts

**Figure 2:**
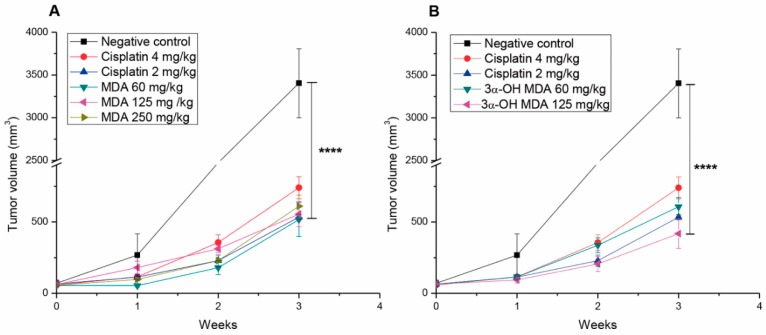
In vivo antitumor effect of masticadienonic and 3α-hydroxymasticadienoic acids. The antitumor activities of MDA (**A**) and 3α-OH MDA (**B**) were evaluated in nu/nu mice transplanted with human protate cancercells. The mice were subcutaneously inoculated with 3 × 10^6^ cells on the right flank. Once the tumors reached 50 mm^3^, the mice received the indicated doses of MDA, 3α-OH MDA, cisplatin or vehicle (sesame oil and 5% dimethyl sulfoxide ) at days 0, 7, 14 and 21. Each point represents the average ± SEM of six mice for each experimental group. **** *p* < 0.0001, significant differences compared with the negative control. Data were analyzed by two-way ANOVA following a multiple comparisons test (Tukey test). All the treated groups were statistically different from negative groups.

The tumor growth inhibition of MDA and 3α-OH MDA was evaluated in nude mice transplanted with PC-3 cells. The tumor growth kinetics after three weeks of the treatment with either cisplatin or the two triterpenes is depicted in Figure 2. Tumors from the negative control group grew to an average volume of 3500 mm^3^, while those treated with MDA, 3αOH-MDA or cisplatin grew to an average volume of 500 mm^3^ (Figure 2A,B).

The differences in the growth of the tumors between the negative control group and those treated with the drugs were statistically significant. These data support the proposal of MDA and 3α-OH-MDA as antitumor compounds in vivo. 

The body weights of mice from all the groups were monitored. The results showed that the average body weight of the animals treated with MDA or 3α-OH MDA and the untreated mice did not change significantly. However, the mice that received cisplatin lost up to 29% of body weight (Table 2).

Evaluation of the preclinical data for systemic toxicity (Table 3) indicated that both MDA and 3α-OH MDA at the administered doses did not produce any significant change in the levels of serum hepatic enzymes and renal parameters such as blood urea nitrogen and creatinine. However, an increase in the liver enzyme, aspartate transaminase (AST) and some renal parameters like creatinine was observed in the cisplatin-treated group. Furthermore, hematological parameters including leukocyte, red blood cell, and lymphocyte counts did not change in the groups treated with the triterpenes. These data suggest that MDA and 3α-OH-MDA were less toxic to the animals than cisplatin.

### 2.4. Histological and Immunohistochemical Analysis of Tumor Tissues

**Figure 3:**
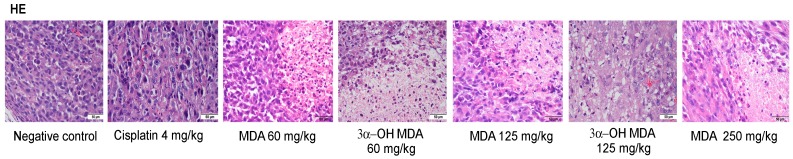
Representative photomicrographs of hematoxylin-eosin staining of tumor tissues.

To evaluate whether the administration of MDA or 3α-OH MDA in the tumor tissues induced changes in cellular morphology, hematoxylin and eosin staining was performed. Sections of PC-3 xenografts from mice treated with MDA or 3α-OH MDA showed that the hematoxylin staining in the cells was markedly diminished in comparison with the negative control (Figure 3). The apoptotic regions were identified by their amorphous shapes and condensed nuclei. Characteristic features of apoptosis were also observed with the TUNEL assay.

**Figure 4:**
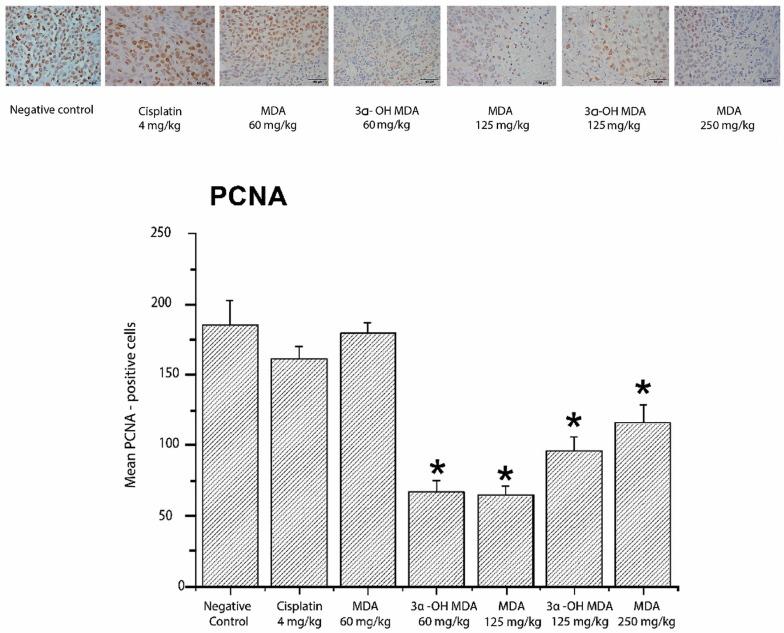
IHC analysis for the proliferating cell nuclear antigen (PCNA) in tumor tissues from mice treated with MDA or 3α-OH MDA for 21 days. The images in Figure 3, Figure 4, Figure 5 and Figure 6 were acquired at 40× using an acquisition software of images (Nis Elements-F). The values across the histogram represent the result of quantitative analysis of PCNA-positive cells. The data are shown as the means ± SEM of three xenografts analyzed for each group. * *p* < 0.05, significant differences compared with the control.

**Figure 5:**
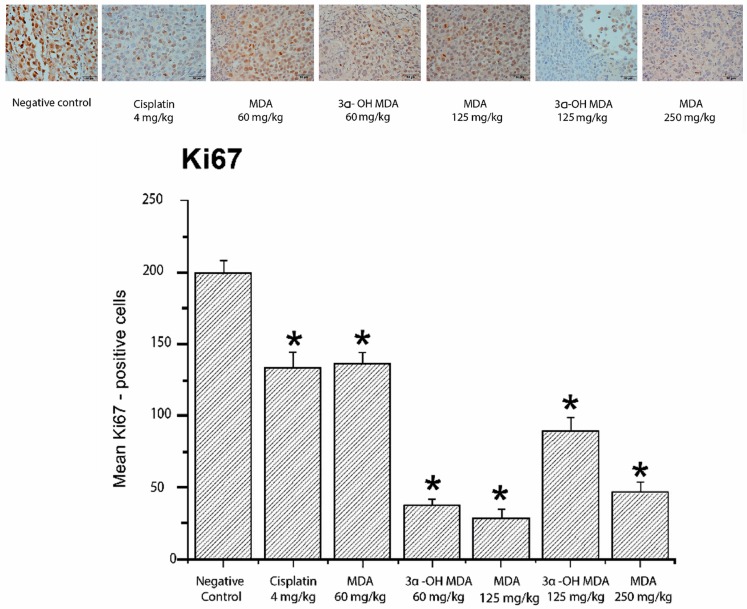
MDA and 3α-OH MDA inhibit the expression of proliferation marker Ki67. Quantitative analysis of Ki67-positive cells was performed by using Image-Pro Plus 7.0 software (Media Cybernetics Inc., Rockville, MD, USA). The data are shown as the means ± SEM of three xenografts analyzed for each group. * *p* < 0.05, significant differences compared with the control.

The inhibition of cell proliferation induced by MDA and 3α-OH MDA was evaluated by the expression of two markers of cell proliferation, proliferating cell nuclear antigen (PCNA) and Ki67. MDA at doses of 125 and 250 mg/kg decreased the average number of cells positive for PCNA and downregulated the expression of Ki67 at the three tested doses. Significant differences were observed in both Ki67-positive cells (*p* < 0.05) and PCNA-positive cells. Furthermore, the integrative optical density (IOD) values for Ki67 were 14.18 ± 1.58, 7.03 ± 1.72 and 6.18 ± 0.96 pixels/µm^2^ (MDA at 60, 125 and 250 mg/kg, respectively) and 3.69 ± 1.23 and 3.08 ± 0.52 (3α-OH MDA at 60 and 125 mg/kg, respectively), which were significantly lower than that for the control group (IOD = 23.37 ± 1.58 pixels/µm^2^). For PCNA, the IOD values were 11.91 ± 0.33, 2.90 ± 0.09 and 9.88 ± 0.52 (MDA at 60, 125 and 250 mg/kg, respectively) and 5.21 ± 0.43 and 7.33 ± 0.29 (3α-OH MDA at 60 and 125 mg/kg, respectively) compared to the IOD of 15.98 ± 0.67 pixels/µm^2^ for the control group on day 21 after treatment. These results indicated that the tumor tissues expressed both PCNA and Ki67 and the expression of these proteins diminished in the tumors treated with MDA or 3α-OH MDA (Figure 4 and Figure 5). Consistent with the microscopic observations in the photomicrographs, we found a quantitative decrease in the number of PCNA-positive and Ki67-positive cells.

**Figure 6:**
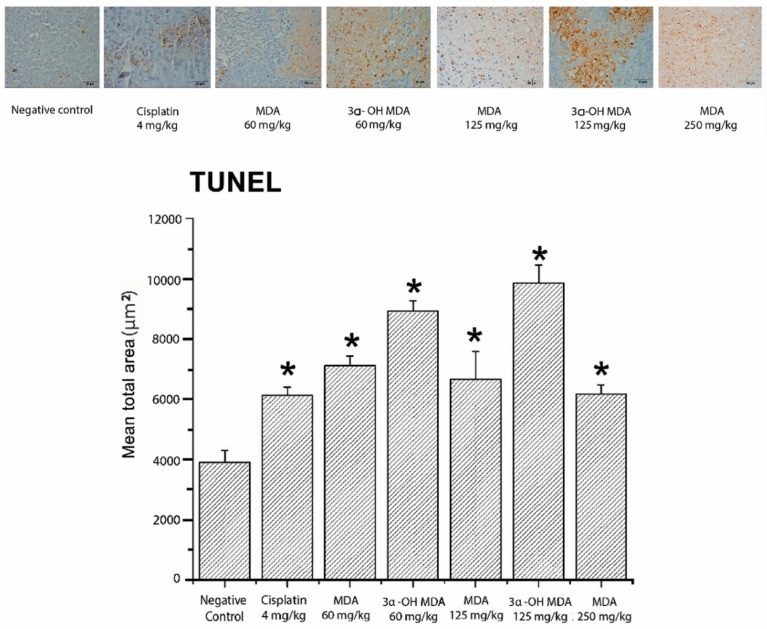
Terminal deoxynucleotidyl transferase dUTP nick end labeling (TUNEL) analysis for the detection of apoptosis in the tumor samples. The values across the histogram represent the result of quantitative analysis of marked areas using Image-Pro Plus 7.0 software (Media Cybernetics). The data are shown as the means ± SEM of three xenografts analyzed for each group. * *p* < 0.05, significant differences compared with the control.

DNA fragmentation by TUNEL assay was investigated as the evidence of apoptotic cell death. The sections of tumors from the MDA- or 3α-OH MDA-treated mice showed numerous apoptotic cells (stained in brown, Figure 6) in comparison with the control group. In all treatments, apoptotic bodies were quantified as a sum of the brown areas. In the case of MDA, the values were 7112.96 ± 326.35, 6668.73 ± 920.09, and 6171.04 ± 296.37 µm^2^ for the doses of 60, 125, and 250 mg/kg, respectively; for 3α-OH MDA, the values were 8919.03 ± 362.49 and 9862.86 ± 601.45 µm^2^ for 60 and 125 mg/kg, respectively; and for the negative control the value was 3891.89 ± 398.20 µm^2^, which resulted in significant differences (*p* < 0.05) (Figure 6).

## 3. Discussion

The number of in vivo preclinical studies on antitumor properties of triterpenes, secondary metabolites with hydrocarbon skeletons of 30 carbon atoms, have increased [16,17,18]. Although there are several reasons for this rise, two of the most prominent reasons include the ease of isolation of some of these compounds and the excellent in vivo antitumor properties of several anti-inflammatory triterpenes despite modest in vitro cytotoxic properties [19].

In our previous work, we reported the anti-inflammatory and cytotoxic properties of MDA and 3α-OH MDA, and the capacity of the 3α-OH MDA to impair mitochondrial functions. However, their cytotoxic effect was depicted with an inhibitory concentration 50% (IC_50_) range between 40 and 70 µM. To supplement the data from the literature, we evaluated the cytotoxic effects of these triterpenes in PC3 cells. As expected, MDA and 3α-OH MDA showed a moderate activity against this cancer cell line (see experimental). Although it is not a formal rule, several authors, journals and the National Cancer Institute (NCI) consider compounds isolated from medicinal plants as active compounds only when they have ED_50_ (effective dose 50) values of ≤ 4 µg/mL [20]. 

Considering the above findings, MDA and 3α-OH MDA would be considered as inactive compounds. Nevertheless, in this work, we investigated the in vivo effect of the two triterpenes in a xenograft model using PC3 cancer cells. Our results showed that MDA and 3α-OH MDA significantly suppressed tumor growth (Figure 2). Tumor suppression was measurable from the fourteenth day of treatment in a 21-day experiment. 

The toxicity of cisplatin was evident due to the loss of body weight in mice at dose of 4 mg/kg (Table 2). Moreover, the mice treated with cisplatin presented with leukopenia, lymphopenia and increased levels aspartate transaminase (AST) at the dose of 2 mg/kg (Table 3). Also in the cisplatin-treated groups, a significant increase in creatinine levels was observed, consistent with previous reports [21]. It is noteworthy that although MDA and 3α-OH MDA decreased tumor growth, both triterpenes did not induce statistical body weight loss (Table 2). 

According to the LD_50_ values, 3α-OH MDA appeared to be more toxic than MDA. This difference in the pharmacological behavior of the two compounds was not expected, since the only structural difference between them is the presence of a carbonyl group at C-3 in MDA (Figure 1).

Regarding the mechanism of action of MDA and 3α-OH MDA, our findings indicate that the intraperitoneal administration of these triterpenes decreased the levels of the proliferation markers PCNA and Ki-67 in the tumor tissues. PCNA is a nuclear non-histone protein that is necessary for DNA synthesis. The critical involvement of PCNA in cellular proliferation and its tight association with transformation in cancer have resulted in its significant importance and application in the clinic [22]. Ki-67 is a classical marker of cellular proliferation. This antigen is preferentially expressed during the late G1, S, G2, and M phases of the cell cycle, whereas resting, non-cycling cells (G0 phase) lack Ki-67 expression [23]. 

MDA and 3α-OH MDA depleted the expression of PCNA (Figure 4) and Ki67 (Figure 5), which indicated their anti-proliferative effect. These results agree with previous studies where anti-proliferative effects of the triterpenes acetyl-11-keto-beta-boswellic acid (AKBA) and nimbolide [8,24] were investigated. 

Terminal deoxynucleotidyl transferase dUTP nick end labeling (TUNEL) assay was used to detect the induction of apoptotic cell death by MDA and 3α-OH MDA. The results showed that both triterpenes are pro-apoptotic (Figure 6). Our findings indicate the likelihood of the following occurrence: membrane permeability transition (MPT) causes a dissipation of the electrical transmembrane potential by matrix swelling and outer membrane disruption, leading to the release of caspase activators, such cytochrome *c* (cyt *c*) and apoptosis-inducing factor (AIF). We recently published that at low concentrations, 3α-OH MDA acts as an intrinsic pro-apoptotic agent, promoting the MPT process and the consequent release of pro-apoptotic factors. In contrast, at a higher concentration, 3α-OH MDA protects rat liver mitochondria against MPT by blocking Ca^2+^ entry [13]. Although it is not always possible to relate the in vitro results with those obtained in vivo, in this study, 3α-OH MDA demonstrated pro-apoptotic activity.

## 4. Materials and Methods

### 4.1. Drugs and Reagents

#### 4.1.1. Media

Roswell Park Memorial Institute medium (RPMI-1640, BIO-L500-500) and FBS (fetal bovine serum, BIO-S1650-500) and Trypsin (BIO-L0931-100) were obtained from Biowest company (Riverside, MO, USA). Dimethyl sulfoxide (D4540-100), Poly-l-lisyne (P8920-100) and Cisplatin (*cis*-Diammineplatinum (II) dichloride, 479306-1G) were obtained from Sigma-Aldrich (St. Louis, MO, USA). TUNEL staining kit was acquired from Promega Corporation (Madison, WI, USA). Proteinase K (IB05406) was obtained from IBI Scientific (Kapp Ct, IA, USA). The antibodies anti-PCNA (sc-9857) and Ki67 (sc-15402) were obtained from Santa Cruz Biotechnology (Dallas, TX, USA). Crystal Violet reagent was acquired from Roche (Mannheim, Germany).

#### 4.1.2. Dissolutions

High-quality water employed to prepare solutions was obtained through a Milli-Q Reagent Water System (Billerica, MA, USA). Stock dissolutions of cisplatin were prepared in saline solution (1 mg/mL) for in vivo studies, and 3 mM for in vitro assays. MDA and 3α-OH MDA (25 mg/mL) were dissolved in extra virgin sesame oil plus 5% of DMSO. Stock solutions of MDA and 3α-OH MDA (22 mM) were prepared in DMSO for in vitro assays, and stored at 4 °C.

#### 4.1.3. Isolation of Masticadienonic (MDA) and 3α-OH Masticadienoic (3α-OH MDA) Acids

Masticadienonic and 3α-OH masticadienoic acids were isolated from *Amphipterygium adstringens*, as previously reported [11].

### 4.2. Acute Toxicity Test

The toxicity assay was performed in 6- to 8-week-old CD-1 female mice. Initially, 3 mice groups were treated with 10, 100 and 1000 mg/kg doses of each triterpene. The treatments were administered intraperitoneally with single injections. Food and water were given up to 4 h after the treatment. Mortality was observed during the first 4 h. Changes in body weight were monitored in the surviving mice for 14 days. At the end of the experiment, the mice were euthanized.

### 4.3. Cell Culture

The human prostate cancer cell line PC-3 was obtained from the American Type Culture Collection (ATCC). The cells were routinely maintained as a monolayer in RPMI-1640 medium supplemented with 10% inactivated fetal bovine serum (FBS), 250 µg/mL streptomycin sulfate, 250 U/mL penicillin, 0.625 µg/mL amphotericin B and 2 mmol L-glutamine and incubated at 37 °C in a 5% CO_2_ atmosphere until 80% confluent. The cells were harvested with 0.025% Trypsin and 1 mmol ethylenediaminetetraacetic acid (EDTA) and washed in phosphate buffered saline (PBS) by centrifugation. After centrifugation, the supernatant was eliminated and the cell pellet was suspended in RPMI-1640 without phenol red and fetal bovine serum.

### 4.4. Determination of Cell Proliferation by Crystal Violet Staining

Cytotoxicity assays were performed by seeding cells in 48-well plates at a density of 4 × 10^4^ cells/cm^2^ in RPMI-1640 phenol red supplemented with 10% FBS at the same culture conditions indicated in 2.3. One day later, MDA or 3α-OH MDA were added at concentrations of 0, 12.5, 25, 50 and 100 µM. Cell viability was evaluated 48 h after the treatment with crystal violet staining. The compounds were dissolved in DMSO to make a stock solution and diluted in RPMI-1640 phenol red supplemented with 10% FBS. Cisplatin was used as the drug reference. After the incubation with MDA or 3α-OH MDA, the adherent cell cultures were fixed by adding 200 µL of glutaraldehyde 1.1% (*w*/*v*) in RPMI-1640 phenol red supplemented with 2% of FBS and incubated at room temperature for 15 min. The supernatant was transferred, and the plates were washed with water and left to air dry. The fixed cells were stained with 200 µL of crystal violet for 15 min, and the protein-bound dye was solubilized with 200 µL of 10% acetic acid (*w*/*v*). The optical density was measured with a microplate reader (Elx808; BioTek Instruments, Inc., Winooski, VT, USA) at 595 nm. A dose-response curve was plotted for each compound, and then IC_50_ was estimated.

### 4.5. Human Prostate Tumor Xenografts

Male nude mice (*nu*/*nu*) aged 6–8 weeks were obtained from the Instituto Nacional de Ciencias Médicas y Nutrición Salvador Zubirán (INCMNSZ), Mexico City, Mexico. The animals were housed under pathogen-free conditions in accordance with Institutional Animal Care Guidelines. All experimental procedures involving animals were approved by the Committee of Animals Research (protocol number 1488) and carried out in accordance to the Guidelines for Care and Use of Laboratory Animals of the INCMNSZ, Mexico City, Mexico. The mice were fed a regular chow diet and had free access to sterile-water.

Groups of six animals were implanted with 3 × 10^6^ PC-3 cells. The cells were subcutaneously inoculated in a 0.1 mL volume of medium (RPMI-1640 without phenol red and fetal bovine serum) into the right flank of the mice. Three weeks after the cell inoculation, the animals were pair-matched into treatment and control groups. The treatments were performed by single weekly intraperitoneal injections of the triterpenes and drug reference. The mice received either MDA (60, 125 or 250 mg/kg) or 3α-OH MDA (60 or 125 mg/kg) dissolved in sesame oil plus 5% DMSO at 0, 7, 14 and 21 days. The positive control group received cisplatin at doses of 2 or 4 mg/kg and the negative control received the vehicle (sesame oil and 5% DMSO). The mice were weighed every week. Tumor size was measured by using a digital Vernier caliper once a week. Tumor volume was determined with the following formula: V = π/6 × (large diameter × [small diameter] ^2^) [25]. After each drug administration, the mice were weighed, and the tumor volume was calculated. After 21 days of treatment, the animals were weighed and anesthetized (sodium pentobarbital), and blood samples were collected by cardiac puncture. Serum was separated by centrifugation (1500 rpm) for further analysis. Determination of biochemical parameters was carried out by the Departamento de Patología de la Facultad de Medicina Veterinaria y Zootecnia, UNAM.

### 4.6. Immunohistochemical Analysis of PCNA and Ki-67

Once the treatment was over, the mice were humanely sacrificed. The tumor tissues were dissected, fixed in 10% formaldehyde, paraffin-embedded and serially sectioned into 4 µm-thick sections. The sections were placed on glass slides coated with 0.1% poly-l-lysine for immunohistochemical analyses (IHC). The tissue sections were first stained with hematoxylin and eosin to determine their histological characteristics. Then, the slides were dewaxed with xylene, gradually hydrated with gradient alcohol (100 to 70%), and washed with PBS. Subsequently, the tissue sections were subjected to heat-induced epitope retrieval by treatment with sodium citrate buffer (pH 6.0). The slides were treated with 30% solution H_2_O_2_ for 10 min at room temperature to block endogenous peroxidase. After rinsing with phosphate-buffered saline (PBS), the slides were blocked with 5% bovine serum albumin (BSA) in PBS for 10 min at room temperature; afterwards, they were incubated at 4 °C overnight with the primary antibodies against PCNA (1:200) and Ki-67 (1:100). After washing with PBS, each slide was incubated with the specific biotinylated secondary antibody for 60 min at 37 °C. Then, the slides were incubated with streptavidin-peroxidase complex for 30 min at 37 °C. The immunoreactive products were visualized by reaction with 3,3-diaminebenzidine (DAB) and hematoxylin counterstain. The negative controls were incubated with albumin solution instead of primary antibodies.

### 4.7. TUNEL Assay

The apoptotic cells in the tissue samples were detected using a Promega kit (G7131) for in situ detection according to the manufacturer’s instructions. The tissues samples were dewaxed and hydrated as described in the previous section. Then, the slides were incubated with proteinase K (1:500) for 15 min at room temperature. The samples were then incubated with the TUNEL reaction mixture, terminal nucleotidyltransferase (rTdT) and biotinylated-dUTP for 60 min at 37 °C. After being washed three times with PBS, the slides were incubated with streptavidin-peroxidase complex for 30 min at 37 °C. The apoptosis signals were visualized with diaminobenzidine. The sections were counterstained with hematoxylin, mounted with mounting medium and analyzed by light microscopy (Nikon Eclipse 80i, Nikon Co., Tokyo, Japan). The negative control was prepared with equilibrium buffer, nucleotide mix, and deionized water without rTdT.

### 4.8. Analysis of Photomicrographs

Photomicrographs of the slides were captured using a Nikon Eclipse 80i microscope equipped with a Nikon DS-U2 camera and by using Nis Elements-F acquisition software (Nikon Co, Tokyo, Japan, version 3.0). Images were digitized at 2560 × 1920 pixels. The images were acquired at 40× magnification and processed in TIF format.

The brown color corresponding to the immunostaining on the slides was quantified as the sum area or nuclei number and Integrative Optical Density (IOD) with Image-Pro Plus 7.0 software (Media Cybernetics, Rockville, MD, USA). First, a spatial calibration of 50 µm was done for each image and the ranges of area and OD were defined in Image-Pro Plus 7.0. Second, the color corresponding to the immunohistochemical signal was segmented according to the Hue, Saturation, Intensity (HSI) model. For a better representation of the assessment of expression levels, a total of 10 random images were acquired for each slide and analyzed using Image-Pro Plus 7.0 software as described in the technical manual with some modifications [26].

### 4.9. Statistical Analysis

Tumor volumes, body weights, number of positive cells (for Ki67 or PCNA) and values of biochemical parameters are reported as the means ± SEM (standard error of mean). Statistical comparisons between drug treatment and the untreated group were done with the Kolmogorov–Smirnov test when the distribution of the data was determined. The data with Gaussian distribution were analyzed by two-way ANOVA following a multiple comparisons test (Tukey test). The data without Gaussian distribution were analyzed with a nonparametric test (ANOVA of Kruska-Wallis) followed by a multiple comparisons test (Dunn’s test). The significant differences were reported with *p*-values.

## 5. Conclusions

Our results showed that the decrease in the levels of the proliferation markers PCNA and Ki-67 as well as the increase in cell death by apoptosis are related to the tumor growth inhibition induced by MDA and 3α-OH MDA in a mouse xenograft model.

The present results demonstrate the activity of the compounds under study in one particular prostate cancer model. More detailed investigations with breast cancer cell lines will be performed. In addition, the possible use of MDA and 3α-OH MDA as adjuvants in antitumor immunization strategies will be investigated.

## Figures and Tables

**Table 1:** Cytotoxic effect of MDA and 3α-OH MDA in prostate cancer cells (PC-3) at 48 h. The inhibitory concentration 50% (IC_50_) values represent the average of three independent assays. The data are shown as the means ± standard deviation (SD).

Treatment	IC_50_ (µM)
MDA	56.51 ± 2.31
3α-OH MDA	54.52 ± 4.95
Cisplatin	15.56 ± 1.14

**Table 2:** Effect of MDA and 3α-OH MDA on body weight of mice. The data are shown as the means ± SEM (standard error of mean) with *n* = 6 animals for each experimental group. ** *p* < 0.01 represent the statistical difference in body weight change between the beginning and end of treatment. Data were analyzed by one-way ANOVA-repeated measures.

Treatment	Weight (g) Initial	Weight (g) Final
Cisplatin 4 mg/kg	19.61 ± 0.61	13.80 ± 0.82 **
Cisplatin 2 mg/kg	25.13 ± 0.68	21.73 ± 0.93
MDA 60 mg/kg	22.06 ± 0.98	20.96 ± 0.67
MDA 125 mg/kg	23.33 ± 1.01	22.25 ± 1.18
MDA 250 mg/kg	24.23 ± 0.88	24.31 ± 1.09
3α-OH MDA 60 mg/kg	23.90 ± 1.16	21.96 ± 1.05
3α-OH MDA 125 mg/kg	24.76 ± 1.76	22.46 ± 1.82
Control without treatment	22.02 ± 1.45	19.72 ± 1.48

**Table 3:** Evaluation of preclinical systemic toxicities of MDA and 3α-OH MDA in the blood after treatment. The values of biochemical parameters are reported as the means ± SEM.

	Healthy	Negative Control	Cisplatin 2 mg/kg	MDA 60 mg/kg	3α-OH MDA 60 mg/kg
Leucocytes (×10^9^/L)	4.46 ± 0.13	3.73 ± 1.73	1.93 ± 0.12 *	4.56 ± 0.92	3.80 ± 0.35
Lymphocytes (×10^9^/L)	2.83 ± 0.76	2.2 ± 1.17	0.75 ± 0.27 *	2.06 ± 1.28	1.46 ± 0.33
Erythrocytes (×10^12^ /L)	7.66 ± 0.43	4.13 ± 1.27	7.16 ± 0.37	7.16 ± 1.0	8.53 ± 0.68
Hemoglobin (g/L)	149.0 ± 2.0	134 ± 6.08	120.66 ± 9.93	135.33 ± 13.24	153.66 ± 5.60
Glucose (mmol/L)	9.37 ± 0.64	11.25 ± 0.66	7.80 ± 0.86	6.94 ± 1.97	8.39 ± 1.92
Urea (BUN) (mmol/L)	7.93 ± 0.68	7.86 ± 0.78	9.03 ± 0.94	8.46 ± 0.03	8.56 ± 1.04
Creatinine (mmol/L)	37.33 ± 8.95	29.33 ± 1.33	84.0 ± 3.21 ****	24.0 ± 6.02	26.66 ± 0.88
Alanine transaminase ALT (U/L)	52.66 ± 5.81	53 ± 5.50	56.66 ± 6.69	53.33 ± 17.48	51.33 ± 7.42
Aspartate transaminase AST (U/L)	155.33 ± 2.40	143.66 ± 9.35	285.66 ± 13.37 ****	145.33 ± 18.66	164.66 ± 6.76

Values for mice show statistical difference in comparison to the negative control, healthy, MDA and 3α-OH MDA groups (* *p* < 0.05), (**** *p* < 0.0001).
